# Visualization of Tau–Tubulin Interaction in a Living Cell Using Bifluorescence Complementation Technique

**DOI:** 10.3390/ijms19102978

**Published:** 2018-09-29

**Authors:** Seulgi Shin, Sungsu Lim, Hyeanjeong Jeong, Li Ting Kwan, Yun Kyung Kim

**Affiliations:** 1 Korea Institute of Science and Technology (KIST), Brain Science Institute, Convergence Research Center for Diagnosis, Treatment and Care System of Dementia, Seoul 02792, Korea; seulgishin@kist.re.kr (S.S.); sungsulim@kist.re.kr (S.L.); guswjd7979@kist.re.kr (H.J.); lkwan@wellesley.edu (L.T.K.); 2 Department of Life Science, Korea University, Seoul 02841, Korea; 3 Department of Neuroscience, Wellesley College, Wellesley, MA 02481, USA; 4 Division of Bio-Medical Science & Technology, University of Science and Technology (UST), Daejeon 34113, Korea

**Keywords:** tau aggregation, microtubule, tubulin, Bifluorescence Complementation, neurodegeneration

## Abstract

Tau is a neuron-specific microtubule-binding protein that stabilizes microtubules. It is generally thought that highly phosphorylated tau dissociates from microtubules and becomes insoluble aggregates, leading to neuronal degeneration. Due to the implication of tau aggregation in neurodegenerative disorders, including Alzheimer’s disease, great efforts have been made to identify the tau aggregation process. However, tau interaction with tubulin during the aggregation process remains largely unknown. To scrutinize the tau-tubulin interaction, we generated a cell model that enables visualization of the tau-tubulin interaction in a living cell using the Bifluorescence Complementation (BiFC) Technique. Upon diverse chemical stimulation that induced tau pathology, tau-tubulin BiFC cells showed significantly increased levels of BiFC fluorescence, indicating that tau aggregates together with tubulin. Our results suggest that tubulin should be considered as a key component in the tau aggregation process.

## 1. Introduction

Tau is a neuron-specific microtubule-associated protein that promotes microtubule assembly and stabilization in healthy neurons [1,2]. Since neuronal structure and axon stability rely on the cytoskeleton framework built by microtubules, tau is crucial to maintain the structural stability of neurons [3]. Abnormal tau aggregation and microtubule destabilization are key in pathological events in multiple neurodegenerative disorders (i.e., Alzheimer’s disease, frontotemporal dementia with parkinsonism-17 (FTDP-17), corticobasal degeneration, Pick’s disease, etc.) classified as tauopathies [4,5]. Accordingly, great efforts have been made to understand and to reverse or halt tau aggregation processes. Thus far, the majority of research has focused on interactions between tau and microtubules, and relatively little is known about tau’s interactions with unpolymerized tubulin.

**Figure 1:**
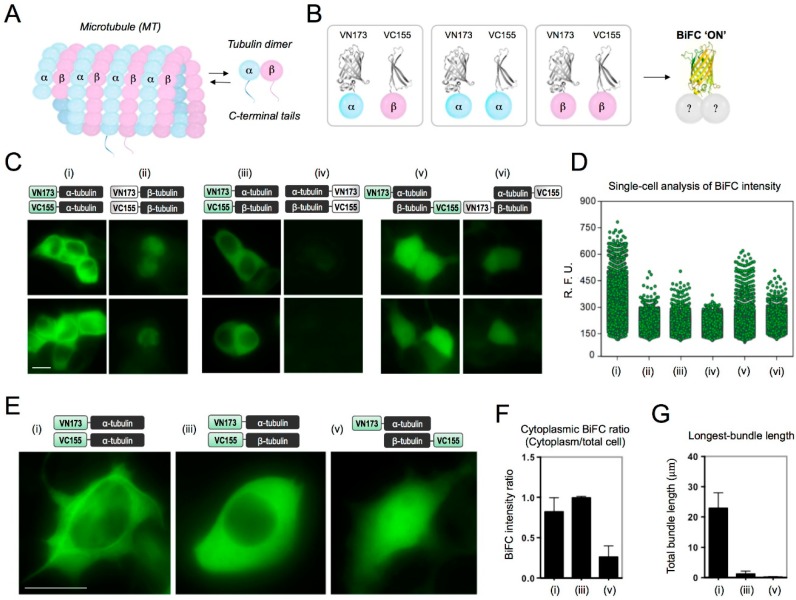
Determination of Bifluorescence Complementation (BiFC) labeling site for α/β-tubulin. (**A**) Structure of a microtubule and α/β-tubulin dimer. (**B**) Diagram of tubulin pairs fused with BiFC compartment. As a fluorescence turn-on sensor, Venus fluorescence turns on upon tubulin-tubulin interaction. (**C**) BiFC fluorescence images of HEK193 cells expressing diverse tubulin-BiFC pairs (i to vi). HEK293 cells were transfected with diverse combinations of α- and β-tubulin-BiFC pairs for 72 h, and were imaged by using Operetta^®^. Scale bar, 20 µm. (**D**) Single-cell analysis of cellular BiFC intensities. BiFC fluorescence intensities were measured using Harmony 3.1 software and plotted using GraphPad Prism software. A total of 19,000 cells were analyzed for each experiment and each dot represents an individual cell. (**E**) High resolution (1000×) images of tubulin-BiFC cells. Scale bar, 20 µm. (**F**,**G**) Quantification of cytoplasmic BiFC fluorescence intensity (**F**) and total bundle length (**G**) of tubulin-BiFC cells. The fluorescence intensities in the cytoplasm or total cell area and total bundle lengths were quantified using Harmony 3.1 software. Data represent the mean ± SD, *n* = 20.

Tau is known to bind to microtubules, which are filamentous structures composed of multiple αβ-tubulin subunits [6] (Figure 1A). Additionally, tau has an affinity to bind to unpolymerized, soluble tubulin [7,8]. A recent study showed that FTDP-17-associated tau mutants have a strong binding affinity with tubulin dimers [9], suggesting that tubulin may play a role in the pathological tau aggregation. In addition, Avila group reported that β-tubulin participates in the formation of tau aggregates in neurofibrillary tangles (NFTs) and Pick bodies [10]. However, most of the previous studies showing tau-tubulin interaction were conducted in vitro using purified tubulin and tau fragments. The in vitro condition has several limitations: first, it prevents the occurrence of post-translational modifications, which are closely associated with the function and dysfunction of tau and tubulin [11,12]; second, in buffer conditions, the formation of the tau-tubulin complex highly depends on the concentrations of tau or tubulin [13]. To scrutinize tau-tubulin interactions under pathological conditions, a cell-based model that can monitor and quantify tau-tubulin interactions in living cells would be a useful tool.

Here, we used the Bifluorescence Complementation (BiFC) Technique to label tau and tubulin. Previously, we demonstrated the usefulness of the BiFC technique to investigate the activation of tau pathology in living cells [14]. Venus fluorescence protein is split into two non-fluorescent N- and C-terminal fragments (VN173 and VC155) and this was used to label tau. As a fluorescence “turn-on” sensor, Venus fluorescence is only activated when tau assembles together in response to diverse chemical and environmental stimuli that trigger tau pathology [15,16,17]. In this study, we generated the tau-tubulin BiFC cell line to visualize tau-tubulin interaction in a living cell and investigated tau-tubulin interaction upon the activation of tau pathology.

## 2. Results and Discussion

### 2.1. Determination of BiFC-Labeling Site for Tubulin

Prior to generating tau-tubulin BiFC constructs, we checked the effect of BiFC-labeling on tubulin; whether BiFC-labeling hinders physiological function of tubulin or cell viability (Figure 1B). Diverse α-and β-tubulin constructs containing VN173 or VC155 fragments at N- or C-terminal were prepared (Figure S1A) and transfected to HEK293 cells in diverse combinations (Figure 1C and Figure S1D). The mRNA expression levels of each tubulin-BiFC pair were cautiously compared for all experiments (Figure S1B,C). Among all combinations, the α-tubulin-BiFC pair showed the strongest BiFC fluorescence (Figure 1D). The high-resolution image of the α-tubulin-BiFC showed that the BiFC fluorescence signal localized on microtubule-bundle structures in the cytosol (Figure 1E(i)). In contrast, the expression of β-tubulin-BiFC pair caused toxicity to the cells, as shown by the shrunken cell morphology. The coexpression of the α- and β-tubulin pairs labeled with BiFC at diverse positions did not show microtubule-like BiFC phenotypes. Our results correspond to previous research showing that the overexpression of β-tubulin, even in small amounts, interrupts microtubule assembly, and ultimately leads to cytotoxicity [18]. Interestingly, when the α- and β-tubulin pair was labeled with BiFC at the N-terminal, enriched BiFC fluorescence was observed in the cytosol (iii), indicating the formation of cytosolic α- and β-tubulin dimers or oligomers (Figure 1F,G). When the α- and β-tubulin pair was labeled with BiFC at the C-terminal (iv), a slightly increased BiFC fluorescence was detected. This result corresponds to previous research that reported that C-terminal labeled tubulin is extremely cytotoxic [19].

When the α- and β-tubulin pair was labeled with BiFC at the N- and C-terminal respectively (v), BiFC fluorescence signal was detected throughout the whole cell, including the nucleus, which is similar to that of the BiFC background fluorescence phenotype (Figure 1E and Figure S1D). Our results clearly indicate that N-terminal BiFC labeling of α-tubulin would be the best position to investigate tau-tubulin interaction while least affecting tubulin function.

### 2.2. Establishment of a Stable Tau-Tubulin BiFC Cell Line

**Figure 2:**
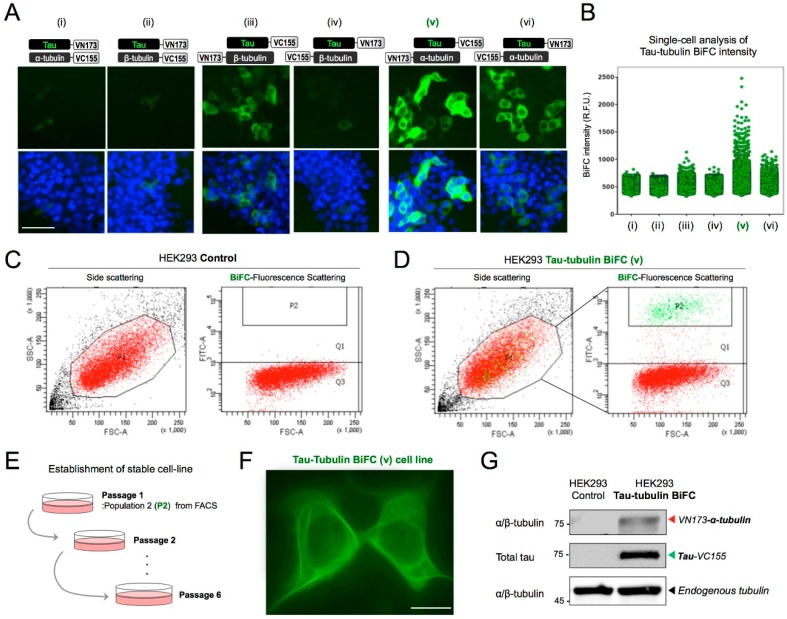
Establishment of the tau-tubulin BiFC cell line. (**A**) BiFC fluorescence images of HEK293 cells transfected with diverse tau- and tubulin-BiFC pairs (i to vi). Scale bar, 50 µm. (**B**) Single-cell analysis of cellular BiFC intensities. A total of 7000 cells were analyzed for each experiment and each dot represents an individual cell. (**C**,**D**) Fluorescence-activated cell sorting (FACS) analysis of tau-tubulin BiFC cells. The top 10% of cells exhibiting the highest BiFC fluorescence intensities were sorted (green dots in P2). HEK293 cells were used as a control. (**E**) Experimental scheme for the generation of tau-tubulin BiFC stable cell line. FACS sorted P2 cells were maintained in a growth medium containing G418 for selection and was passaged 6 times. (**F**) High resolution (1000×) fluorescence image of tau-tubulin BiFC cells showing tau and tubulin interaction in live cells. Scale bar, 20 µm. (**G**) Immunoblot analysis of tau-tubulin BiFC cells with tau (S262) and α/β-tubulin antibodies; HEK293 cells were used as a control.

To screen tau-tubulin BiFC pairs, HEK293 cells were transfected with six different combinations of tau-tubulin BiFC constructs (Figure 2A(i–vi)). The mRNA expression levels of each tau-tubulin pair were compared (Figure S2A,B). As expected, the most significantly increased BiFC-intensity was observed in cells expressing N-terminal labeled α-tubulin and C-terminal labeled tau (v). Again, when the α- or β-tubulin pair was labeled at C-terminal (i, ii), or when β-tubulin was labeled, BiFC-fluorescence was slightly increased (Figure 2B).

To generate a stable cell line, HEK293 expressing Tau-VC155 and VN173-tubulin-α (v) were sorted using fluorescence-activated cell sorting (FACS). The top 10% of cells (P2) showing the highest BiFC fluorescence intensity were collected (Figure 2D). Figure 2C represents HEK293 control cells, which did not show any BiFC fluorescence. The collected cells were cultivated in a growth medium containing the selective agent G418 (Figure 2E). After six cell passages, the tau-tubulin BiFC cell line was stabilized and showed a microtubule bundle-like BiFC fluorescence phenotype (Figure 2F). An immuno-blot analysis of tubulin and tau was followed to verify the expression of α-tubulin and tau (Figure 2G). VN173-tubulin-α and tau-VC155 were detected near 75 kD. The stable cell line was named tau-tubulin BiFC and used to investigate tau-tubulin interaction.

### 2.3. Live-Cell Investigation of Tau–Tubulin Interaction upon the Treatment with Microtubule-Disrupting Agents

**Figure 3:**
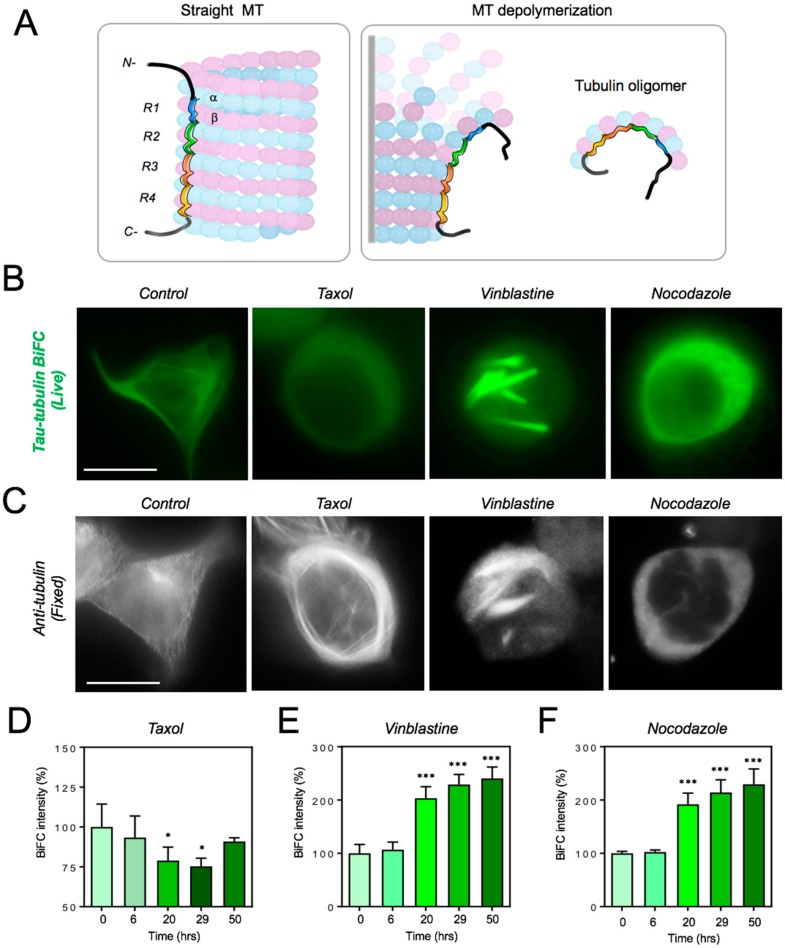
Live-cell investigation of tau-tubulin interaction upon the treatment with microtubule-disrupting agents. (**A**) Diagram of tau-tubulin interaction in a straight microtubule (MT) or in a depolymerized microtubule. (**B**) High-resolution (1000×) BiFC fluorescence images of tau-tubulin BiFC cells upon treatment with taxol, vinblastine, or nocodazole. Scale bar, 20 μm. (**C**) Tubulin immunofluorescence images of tau-tubulin BiFC cells treated with each compound. Scale bar, 20 μm. (**D**–**F**) Quantification of BiFC fluorescence intensities upon treatment with taxol (**D**), vinblastine (**E**), or nocodazole (**F**). BiFC intensities were measured at diverse time points. Data represent the mean ± SD of four independent experiments. The significance of the experiments was determined by Student’s *t*-test. * *p* < 0.05, *** *p* < 0.001 compared to time 0.

According to a recent study, four repeat domains of tau bind to the microtubule surface in tandem, which support inter- and intra-dimer interface along a microtubule or a tubulin oligomer [20] (Figure 3A). To observe changes in tau-tubulin interaction, we treated tau-tubulin BiFC cells with microtubule dynamic disrupting agents. First, we used taxol, which is known to stabilize microtubules by binding to the N-terminus of β-tubulin [21,22,23,24]. It is also known that tau competes with taxol for the same binding sites on microtubules [25,26]. Upon treatment with taxol, tau-tubulin interaction was noticeably decreased, as shown by a 25% reduction in BiFC intensity (Figure 3B,D). An immunofluorescence stain of tubulin showed extensively enriched microtubule bundles in the cytoplasm (Figure 3C). This result indicates that taxol treatment shifted the equilibrium of tubulin from the soluble to polymerized form and dissociated tau from microtubules.

Next, we treated the tau-tubulin BiFC cells with vinblastine, which is known to inhibit microtubule assembly and to induce tubulin self-association into coiled spiral aggregates [27,28,29]. Upon treatment with vinblastine, BiFC intensity increased by 2.3 times and cells showed spiral filament phenotypes (Figure 3B,C,E). An immunofluorescence stain of tubulin also showed spiral filament structures (Figure 3C). Our results clearly indicate that tau-tubulin interaction increases upon treatment with vinblastine. The result contradicts recent observations by Kadavath et al. in an in vitro binding assay using purified tubulin and tau fragments that tau-tubulin interaction is impeded by vinblastine [7]. The contrasting result might have been caused by the differences between an in vitro binding assay and a live-cell investigation.

Next, tau-tubulin BiFC cells were treated with Nocodazole, which interferes with microtubule polymerization [30,31,32]. Upon treatment with nocodazole, microtubules were depolymerized (Figure 3C). Conversely, BiFC fluorescence intensity increased by 2.4 times in the cytosol (Figure 3B,F). This result suggests that tau strongly binds to cytosolic tubulin oligomers, which is increased by nocodazole treatment. Several in vitro studies reported that tau binds to free cytosolic tubulin dimers and enhances microtubule polymerization [9,13]. Our results confirm that tau binds to unpolymerized tubulin in the living cell.

### 2.4. Increased Tau–Tubulin Interaction upon the Treatment of Tau Aggregation Inducters

Next, we investigated tau–tubulin interaction under pathological tau aggregation inducing conditions. Tau-tubulin BiFC cells and tau-BiFC cells [14] were treated with well-known tau aggregation inducers: forskolin, an activator of protein kinase A (PKA), is known to induce tau aggregation [14]; thapsigargin, an endoplasmic reticulum (ER)-stress inducer, is known to induce tau hyperphosphorylation [33]; tauK18^P301L^ purified protein, a prion-like tau species, is known to induce intracellular tau aggregation [15].

**Figure 4:**
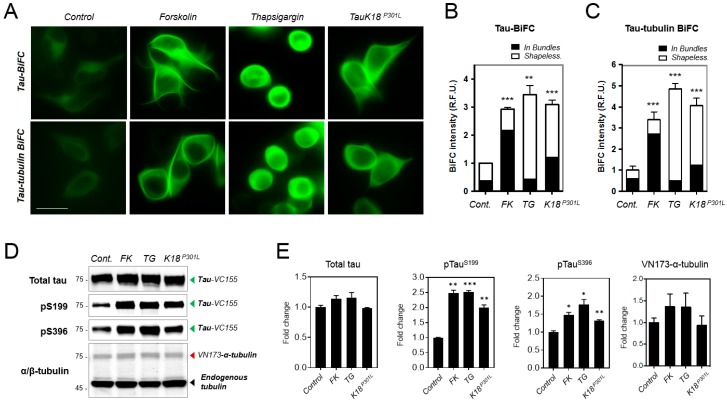
Increased tau-tubulin interaction upon the treatment of tau aggregation inducers (**A**) BiFC fluorescence images of tau-BiFC and tau-tubulin BiFC treated with tau aggregation inducers. Tau-BiFC and tau-tubulin BiFC cells were incubated with forskolin (30 μM), thapsigargin (1 μM), or tauK18^P301L^ (5 μg/mL) for 24 h, and imaged. Scale bar, 20 μm. (**B**,**C**) Quantification of BiFC fluorescence intensities. Total cell area and bundle area were selected, and each BiFC fluorescence intensities were calculated using Harmony 3.1 software. “Shapeless” BiFC fluorescence intensity was calculated by subtracting the bundle intensity from the total cell intensity. Error bars represent the standard deviation of two independent experiments. ** *p* < 0.01, *** *p* < 0.001. (**D**) Immuno-blot analysis of total tau, phosphorylated tau, and α-tubulin with anti-TauS262, p-Tau (S199), p-Tau (S396), or α/β-tubulin antibodies. Endogenous tubulin was used as a loading control. (**E**) Quantification of total tau, phosphorylated tau, and VN173-α-tubulin. The relative amount of VN173-α-tubulin was normalized with that of endogenous tubulin. Data represent the mean ± s.d. of replicate experiments. * *p* < 0.05, ** *p* < 0.01, *** *p* < 0.001 compared to control.

Upon treatment with diverse tau aggregation inducers, in all cases, tau-tubulin BiFC cells showed dramatically increased BiFC intensities, comparable to that of tau-BiFC cells (Figure 4A). Forskolin treatment increased BiFC intensity 2.9-fold in tau-BiFC cells and 3.4-fold in tau-tubulin BiFC cells (Figure 4A,B and Figure S3). A forskolin treatment is known to induce a filament-shaped tau aggregation in tau-BiFC cells [14]. Similarly, forskolin-treated tau-tubulin BiFC cells exhibited a thick bundle phenotype, on which BiFC fluorescence was significantly increased (Figure 4C). Thapsigargin treatment increased BiFC intensity 3.4-fold in tau-BiFC cells and 4.7-fold in tau-tubulin BiFC cells (Figure 4B,C and Figure S3). In the case of thapsigargin, increased BiFC fluorescence was shapelessly exhibited in the cytosol of both tau-BiFC and tau-tubulin BiFC cells. Upon the treatment of prion-like tau seed (tauK18^P301L^), BiFC intensity increased 3.1-fold in tau-BiFC cells and 4.1-fold in tau-tubulin BiFC cells (Figure 4B,C and Figure S3). The increased BiFC signal can be observed from both bundle and shapeless forms. Forskolin treatment increased BiFC fluorescence on bundles both in tau-BiFC and tau-tubulin BiFC cells. Microtubule-immunofluorescence stain also showed a thick bundle of microtubule suggesting that tau-tubulin interaction was strengthened on microtubule (Figure S4). In comparison, thapsigargin treatment increased BiFC fluorescence on shapeless cytosol both in tau-BiFC and tau-tubulin BiFC cells. A microtubule-immunofluorescence stain showed that microtubules disappeared upon thapsigargin treatment (Figure S4). The result suggests that thapsigargin induced tau-tubulin interaction in soluble forms. Immuno-blot analysis of the tau-tubulin total cell lysate showed that pathological tau phosphorylation at Ser199 and Ser396 was induced by the treatment with forskolin, thapsigargin, and tauK18^P301L^ (Figure 4D,E). In addition, pathological tau aggregation in tau-tubulin BiFC cells was confirmed by using a tau aggregation specific fluorescence probe, named BD-tau [34] (Figure S5). Our results strongly suggest that tubulin co-aggregates with tau during pathological tau aggregation process.

### 2.5. Discussion

Neuronal structure and axon stability rely on the cytoskeleton framework built by microtubules, which are polymers that vary in length and are composed of tubulin subunits. In neurons, tau binds to αβ-tubulin subunits in tandem and promotes microtubule assembly. Thus, the relationship between tau and microtubules is important to maintain neuronal stability and function, and its disruption causes neuronal degeneration. Although there is a report showing that β-tubulin is associated with the formation of NFTs and Pick bodies in patients’ brains, the way tau interacts with tubulin under pathological condition remained largely unknown. To investigate tau-tubulin interaction, we developed a tau-tubulin BiFC cell model. By using the cell model, we were able to visualize soluble tau-tubulin complexes induced by nocodazole and spiral shaped tau-tubulin filaments induced by vinblastine in living cells. Additionally, we observed that tubulin co-aggregated with tau under diverse chemical stimuli that induced tau aggregation. Our results strongly suggest that tubulin should be considered as a vital component of pathological aggregates associated with tauopathies.

## 3. Experimental Section

### 3.1. Construction of Tubulin-BiFC Plasmids

α- and β-tubulin-BiFC plasmids were constructed on the basis of the tau-BiFC plasmid (pCMV6-hTau40-VN173 and pCMV6-hTau40-VC155), described in our previously published article [14]. To replace tau with tubulin, α- and β-tubulin cDNAs were purchased from OriGene Technologies, Inc. (NM006009 and NM178014) and amplified using PCR primers containing BglII/PmeI (Tubulin-α) and KpnI/PmeI (Tubulin-β) restriction sequences: (Tubulin-α F) 5′-CTGTACAAGAGATCTATGCGTGAGTGCATCTCCATC-3′, (Tubulin-α R) 5′-CGGCCGGCCGTTTAAACTCATCAATGTATCTTATCATGTCTGGAT-3′, (Tubulin-β F) 5′-CTGTACAAGGGTACCATGAGGGAAATCGTGCACATCCAG-3′, and (Tubulin-β R) 5′-CGGCCGGCCGTTTAAACTCATCAATGTATCTTATCATGTCTGGATCCC-3′. The following sets of α- and β-tubulin-BiFC plasmids were constructed: pCMV6-VN173-Tubulin-α, pCMV6-VC155-Tubulin-α, pCMV6-Tubulin-α-VN173, pCMV6-VN173-Tubulin-β, pCMV6-VC155-Tubulin-β, and pCMV6-Tubulin-β-VN173 (Figure S1A). All plasmid constructs were confirmed through DNA sequencing and restriction enzyme digestion.

### 3.2. Transient Transfection and BiFC-Image Acquisition

HEK293 cells were maintained in Dulbecco’s modified eagle medium (DMEM) containing 10% fetal bovine serum (FBS), 100 units/mL penicillin, and 100 μg/mL streptomycin at 37 °C in a humidified atmosphere containing 5% CO_2_. For the transient transfection, HEK293 cells were plated on μ-clear 96-well plates with an Opti-MEM medium (Invitrogen). After 12 h of cell attachment, the cells were transfected with 0.1 μg each pairs of tubulin-tubulin BiFC or tau-tubulin BiFC plasmids using Lipofectamine^®^2000 reagent (Invitrogen, Waltham, MA, USA). At 48 and 72 h post transfection, the entire plates were automatically imaged by using Operetta^®^ (PerkinElmer, Waltham, MA, USA). All high resolution images were acquired by using a Nikon Eclipse inverted microscope (Ti, Nikon, Tokyo, Japan) at 1000× magnification.

### 3.3. Establishment of Tau-Tubulin BiFC Stable Cell Line

For the generation of tau-tubulin BiFC cell line, the cells were co-transfected with pCMV6-hTau40-VC155 and pCMV6-VN173-Tubulin-α. To establish the stable cell line, the transfected cells were incubated with growth medium containing 100 g/mL Geneticin (G418, Sigma, St. Louis, MO, USA) for selection. Fluorescent cells were then sorted by using FACSAria (BD Bioscience, San Jose, CA, USA) to enrich the population.

### 3.4. Cell Culture and Compound Treatment

Tau-tubulin BiFC and tau-BiFC cells were maintained in DMEM containing 10% FBS, 100 units/mL penicillin, 100 μg/mL streptomycin, 100 μg/mL G418 at 37 °C in a humidified atmosphere containing 5% CO_2_. For treatment with microtubule-disrupting agents, tau-tubulin BiFC cells were plated on μ-clear 96-well plate. The next day, the cells were treated with taxol, nocodazole, and vinblastine at 1 μM concentration. After 6, 20, 29, and 50 h of incubation, the entire 96-well plate was imaged automatically by using Operetta^®^ (PerkinElmer, Waltham, MA, USA).

To induce tau aggreation, tau-BiFC and tau-tubulin BiFC cells were incubated with forskolin (30 µM), thapsigargin (1 µM), or tauK18^P301L^ (5 µg/mL) for 24 h. TauK18^P301L^ fragment was purified from *E. coli* followed by the previously estabilished protocol [15].

The entire 96-well plate was automatically imaged under the same exposure by using Operetta^®^ (PerkinElmer). High resolution images were acquired by using a Nikon Eclipse inverted microscope (Ti, Nikon) at 1000× magnification.

### 3.5. BiFC-Image Analysis

BiFC fluorescence images acquired by using Operetta^®^ were analyzed using the Harmony 3.1 software (PerkinElmer). All experiments were performed in triplicate. The means and standard deviations of BiFC fluorescence intensities were plotted using a Prism software 7 (GraphPad). Quantification data was analyzed by Student’s *t*-test.

### 3.6. Immunofluorescence Stain

For the tubulin-immunofluorescence stain, tau-tubulin BiFC cells grown in a 96-well plate were treated with taxol, nocodazole, or vinblastine for 24 h. Then, cells were fixed with 3.7% paraformaldehyde, and permeabilized with PBS containing 0.1% Triton X-100. For detection of α-tubulin, the primary antibody DM1A (AbCam, 1:1000) was used. High-resolution fluorescence images were acquired using a Nikon Eclipse inverted microscope at 1000× magnification.

### 3.7. Immunoblot Analysis

Tau-tubulin BiFC cells grown in a 6-well plate were treated with forskolin (30 µM), thapsigargin (1 µM), or tauK18^P301L^ (5 µg/mL) for 48 h. Then, cell lysates were prepared with RIPA lysis buffer (Sigma, St. Louis, MO, USA) containing protease and phosphatase inhibitor cocktail (Sigma). Of the protein lysates, 10µg was separated on an SDS-PAGE gel (7.5%) and transferred to PVDF membranes for immuno-blot analysis. Tau antibodies; Ser262, pSer199, pSer396, and α/β-tubulin antibody were purchased from AbCam (Cambridge, MA, USA). Quantification data was analyzed by Student’s *t*-test.

## Supplementary Materials

Supplementary materials can be found at http://www.mdpi.com/1422-0067/19/10/2978/s1.
