# Supplementary material for: Visualization of Tau–Tubulin Interaction in a Living Cell Using Bifluorescence Complementation Technique
Source: Int J Mol Sci. 2018 Sep 29;19(10):2978. doi: 10.3390/ijms19102978 (PMC6213793; doi:10.3390/ijms19102978)
Supplement: Supplementary file 1 [file ijms-19-02978-s001.pdf]

*Int. J. Mol. Sci.* **2018**

OPEN ACCESS
International Journal of
**Molecular Sciences**
**ISSN 1422-0067**
www.mdpi.com/journal/ijms

*Article*

# Visualization of tau-tubulin interaction in a living cell using Bifluorescence Complementation Technique

**Seulgi Shin[1], Sungsu Lim[1], Hyeanjeong Jeong[1,2], Li Ting Kwan [1,3] and Yun Kyung Kim[1,4]***

[1] Korea Institute of Science and Technology (KIST), Brain Science Institute, Convergence Research Center for Diagnosis, Treatment and Care System of Dementia, Seoul, 02792, Republic of Korea; seulgishin@kist.re.kr, sungsulim@kist.re.kr
[2] Department of Life Science, Korea University, Seoul, 02841, Republic of Korea; guswjd7979@kist.re.kr
[3] Wellesley College, Department of Neuroscience, Wellesley, MA 02481, USA; lkwan@wellesley.edu
[4] Division of Bio-Medical Science & Technology, University of Science and Technology (UST), Daejeon, 34113, Republic of Korea

* Correspondence: yunkyungkim@kist.re.kr; Tel.: +82-2-958-5151*

Author to whom correspondence should be addressed; E-Mail: yunkyungkim@kist.re.kr
Tel.: +82-2-958-5072; Fax: +82-2-958-5059.

# Supplementary Information

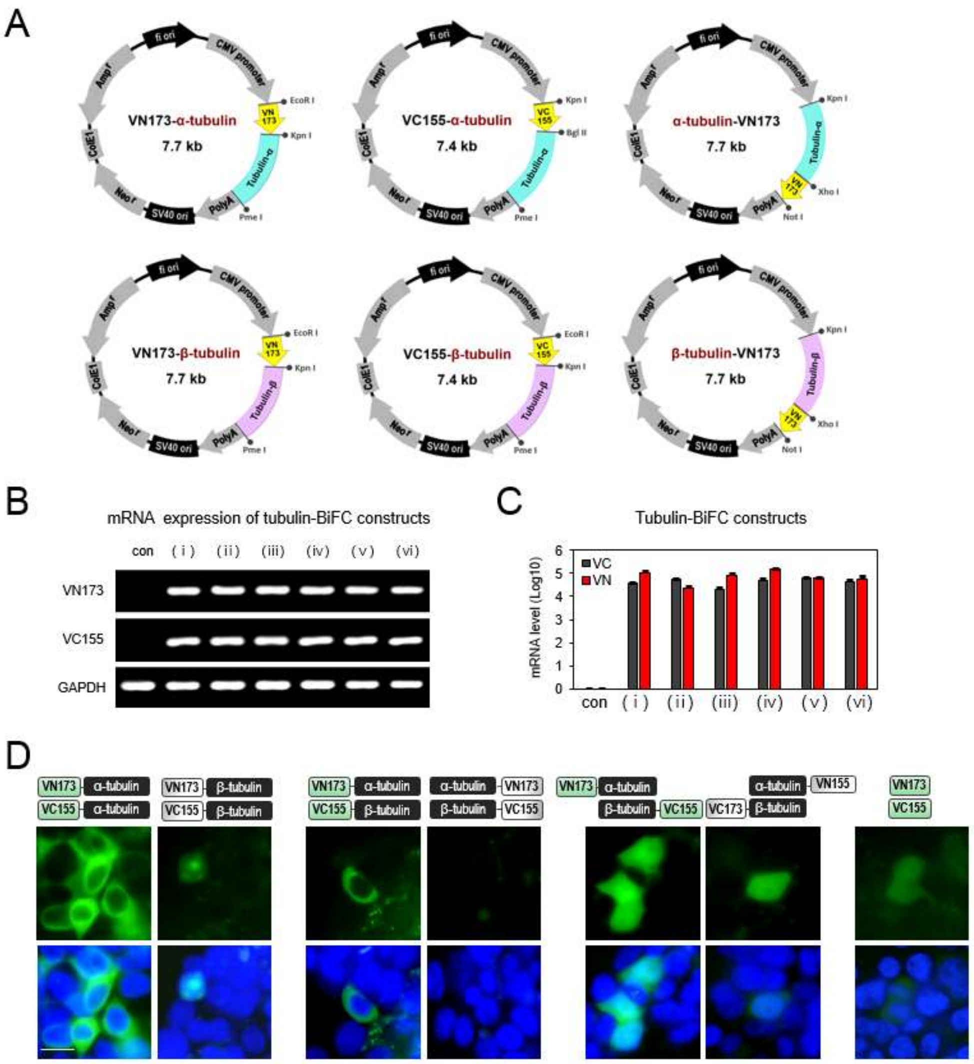


**Supplementary Figure 1. Expression of tubulin-BiFC constructs.** (A) Vector maps of N- or C-terminal BiFC labeled tubulin constructs. (B-C) mRNA expression of BiFC-labeled tubulin constructs. HEK293 cells were transfected with diverse combination of α- and β-tubulin BiFC pairs (i to vi in Figure 1C). 72 hrs after transfection, total RNA was extracted. cDNA synthesized from mRNA was fractionized by gel electrophoresis (B) and analyzed by RT-PCR with VN173, VC155 primers (C). Data represent the mean ± s.d. of replicate experiments. (D) BiFC fluorescence images of HEK193 cells expressing diverse tubulin-BiFC pairs. Nuclei were counterstained with Hoechst. Scale bar, 20 μm.

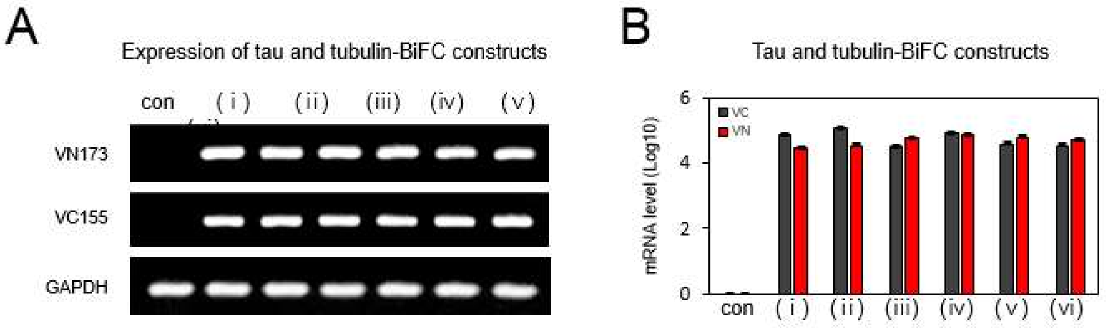


**Supplementary Figure 2. Expression of tau- and tubulin-BiFC constructs.** (A-B) mRNA expression of BiFC-labeled tau and tubulin constructs. HEK293 cells were transfected with diverse combination of tau- and tubulin-BiFC pairs (i to vi in Figure 2A). cDNA synthesized from mRNA was fractionized by gel electrophoresis (B) and analyzed by RT-PCR with VN173, VC155 primers (C). Data represent the mean ± s.d. of replicate experiments.

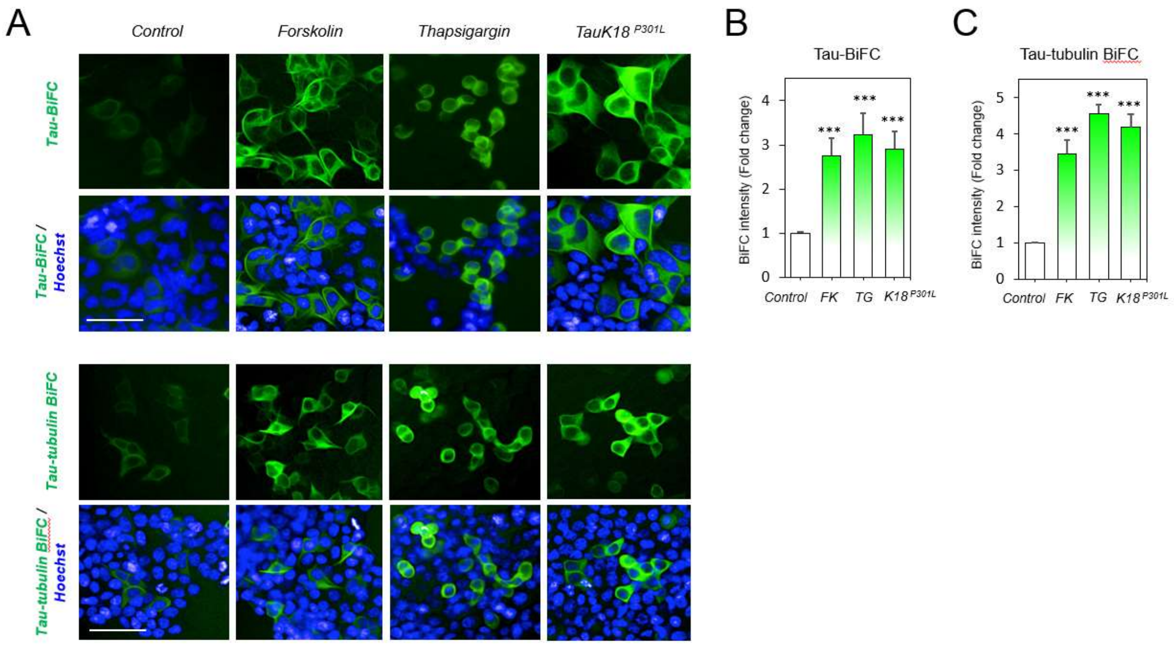


**Supplementary Figure 3. BiFC response upon treatment of tau aggregation inducers.** (A) BiFC fluorescence images of tau-BiFC and tau-tubulin BiFC treated with tau aggregation inducers. Tau-BiFC cells and tau-tubulin-BiFC cells were incubated with forskolin (30 μM), thapsigargin (1 μM), or TauK18$^{P301L}$ (5 μg/ml) for 24 hr, and BiFC fluorescence was imaged by using Operetta®. Nuclei were counterstained with Hoechst. Scale bar, 100 μm. (B,C) Quantification of BiFC fluorescence intensity in tau-BiFC cells (B) and tau-tubulin BiFC cells (B). BiFC intensities were calculated using a Harmony 3.1 software. Data represent the mean ± s.d. of two independent experiments. ***$p < 0.001$.

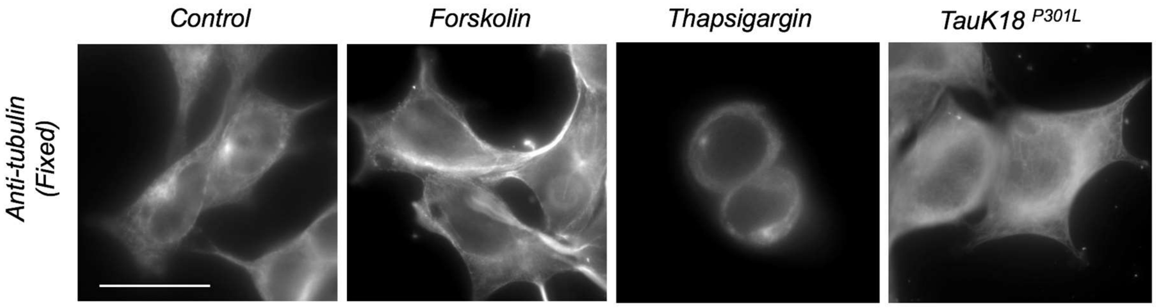


**Supplementary Figure 4. Microtubule-immunofluorescence stain of tau-tubulin BiFC cells.** High-resolution (1,000 X) BiFC fluorescence images of tau-tubulin BiFC cells upon treatment with forskolin, thapsigargin, or tauK18$^{P301L}$. Scale bar, 20 μm. Upon the Forskolin treatment, a thick bundle of microtubule was observed. In comparison, microtubule bundles were disappeared upon thapsigargin treatment.

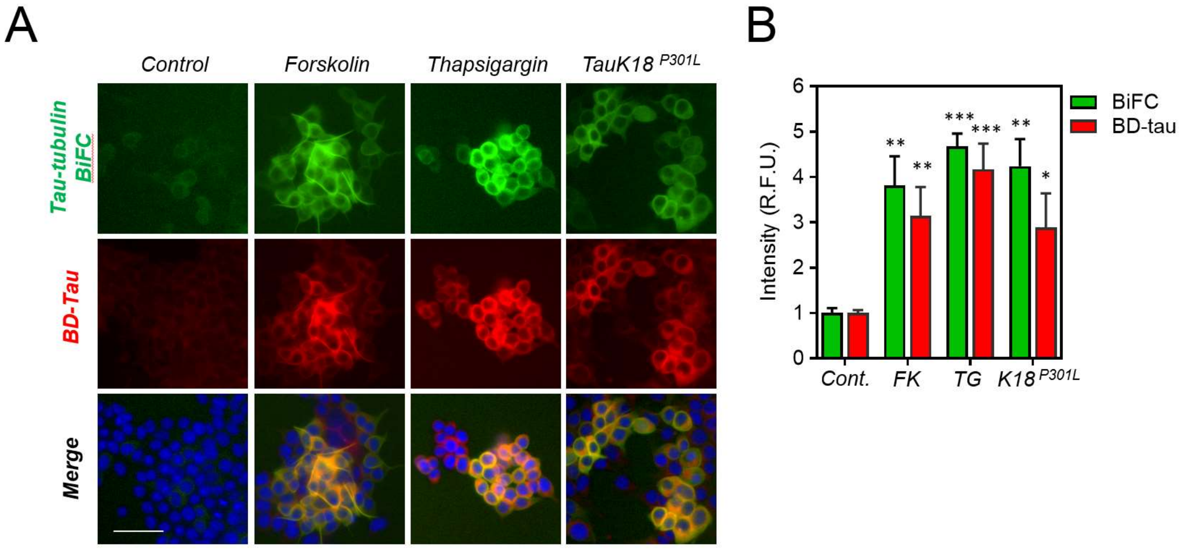


**Supplementary Figure 5. BD-tau stains pathological tau aggregation in tau-tubulin BiFC cells** (A) Tau-tubulin BiFC cells treated with tau aggregation inducers. Tau-tubulin-BiFC cells were incubated with forskolin (30 μM), thapsigargin (1 μM), or TauK18$^{P301L}$ (5 μg/ml) for 48 hr, and treated with 300 nM BD-tau for 30 mins. BiFC fluorescence images and BD-tau fluorescence images were taken by using Operetta®. Nuclei were counterstained with Hoechst. Scale bar, 50 μm. (B) Quantification of BiFC fluorescence intensity and BD-tau intensity. BiFC and BD-tau intensities were calculated using a Harmony 3.1 software. Data represent the mean ± s.d. of three independent experiments. *$p < 0.05$, **$p < 0.01$, ***$p < 0.001$ compared to BiFC control or BD-tau control, respectively.

## Supplementary Methods

*RNA extraction and real-time quantitative RT-PCR (QPCR) analysis*

Total RNA was extracted from the transfected cells by using TRIzol reagent (Life Technologies) following the manufacturer's instructions. mRNA transcription was conducted with TaqMan Reverse Transcriptase (Bioneer Inc.), and real-time quantitative polymerase chain reaction (QRCR) was performed with FAST qPCR Kit Master Mix (KAPABIOSYSTEMS). The reaction was amplified, and mRNA quantity was assessed by using StepOne Plus (Life Technologies) according to the manufacturer's instructions. The relative quantity (RQ) of mRNA was obtained through the comparative threshold cycle (Ct) method and normalized using GAPDH as an endogenous control. Primer sequences for VN173, VC155, GAPDH were the are the following; (VN173 F) 5'-GAC GAC GGA CAA CTA CAA GAC-3', (VN173-R) 5'-TTC AGC TCG ATG CGG TTC AC-3', (VC155 F) 5'-AGC AAA GAC CCC AAC GAG AA-3', and (VC155 R) 5'-TCG TCC ATG CCG AGA GTG AT-3', (GAPDH F) 5'-CGC TCT CTG CTC CTC CTG TT-3', (GAPDH R) 5'-CCA TGG TGT CTG AGC GAT GT-3'.
